# Experimental Investigation of Radiation-Shielding, Mechanical and Thermal Properties of Colemanite-Modified Gypsum-Based Composites

**DOI:** 10.3390/ma19122439

**Published:** 2026-06-07

**Authors:** Hayrettin Eroğlu, Hasan Murat Çetin, Felix N. Okonta

**Affiliations:** 1 Department of Chemical Engineering, Faculty of Engineering and Architecture, Erzurum Technical University, Erzurum 25050, Türkiye; 2 Department of Architecture, Building Information Division, Faculty of Architecture and Design, Atatürk University, Erzurum 25240, Türkiye; hasanmurat.cetin@atauni.edu.tr; 3 Department of Civil Engineering Science, University of Johannesburg, Johannesburg 2094, South Africa; fnokonta@uj.ac.za

**Keywords:** colemanite, gypsum composites, radiation shielding, thermal conductivity, microstructure, boron minerals

## Abstract

**Highlights:**

**What are the main findings?**

- Colemanite incorporation enhances X/γ and neutron shielding performance of gypsum composites.
- Optimal gamma-shielding performance is achieved at 2.5–5 wt.% colemanite content.
- Neutron attenuation increases continuously with increasing colemanite/boron content.
- Maximum compressive strength is obtained at 2.5 wt.% colemanite.
- Thermal conductivity reaches its minimum at 5 wt.% colemanite.
- Microstructural analyses show improved homogeneity at optimal additive ratios.
- Excess colemanite (>5 wt.%) leads to agglomeration and reduced mechanical strength.

**What are the implications of the main findings?**

- Colemanite-modified gypsum can be used as a multifunctional shielding material.
- It enables lightweight alternatives to lead-based radiation protection systems.
- It is suitable for medical, nuclear, and laboratory building applications.
- It provides combined radiation-shielding and thermal insulation performance.
- The optimal additive ratio should be selected according to application requirements.
- It offers a cost-effective and environmentally friendly construction solution.
- It supports the design of safer and more sustainable radiation-protective structures.

**Abstract:**

In this study, the radiation-shielding, mechanical, microstructural, and thermal properties of gypsum-based composites modified with thermally treated colemanite were investigated. Composite samples containing 1, 2.5, 5, and 10 wt.% colemanite were prepared, and the additive was pre-treated at 650 °C to improve its stability and compatibility with the matrix. Gamma-ray attenuation was evaluated at different photon energies, and neutron attenuation was determined using a ^241^Am–Be source. The results showed that colemanite addition improves gamma-ray attenuation, particularly at low and medium energies, with the best performance observed at 2.5–5 wt.%. Neutron attenuation increased with an increasing colemanite content due to the presence of boron. Compressive strength exhibited a non-linear trend, reaching its maximum at 2.5 wt.% and decreasing at higher contents due to microstructural changes. Thermal conductivity also showed non-linear behavior, with the lowest value obtained at 5 wt.% colemanite. SEM and FTIR analyses confirmed the incorporation of colemanite and its influence on the microstructure. Overall, the results indicate that colemanite-modified gypsum composites provide a balanced combination of radiation-shielding, thermal insulation, and mechanical performance.

## 1. Introduction

The widespread use of nuclear technologies in energy production, medical imaging, radiotherapy, and various industrial applications has significantly increased the need for materials capable of providing effective protection against ionizing radiation [1,2]. In this context, the development of building materials that exhibit both high mechanical strength and the ability to effectively attenuate ionizing radiation has become an important research topic in the fields of materials science and nuclear engineering [3,4].

Gamma rays are high-energy electromagnetic waves, and their interactions with matter occur mainly through photoelectric effect, Compton scattering, and pair production mechanisms. The probability of these interaction processes varies depending on photon energy, material density, and atomic number [5]. Therefore, materials with high density and high atomic number have traditionally been preferred for gamma radiation-shielding applications. In particular, lead and heavy aggregate concretes provide effective shielding performance due to their high density and atomic number. However, lead has significant limitations such as its toxic nature, environmental risks, processing difficulties, and high cost [2,6]. Similarly, although heavy aggregate concretes offer advantages in terms of high density, they do not provide a suitable solution for every application due to cost, supply difficulties, and increased structural load. This situation has accelerated studies aimed at developing more environmentally friendly, economical, and applicable alternative shielding materials [3,4].

Neutron radiation, on the other hand, has a different interaction mechanism compared to gamma rays due to its neutral structure. The basic approach in neutron shielding is to slow down fast neutrons through elastic collisions in hydrogen-rich environments and then absorb them using elements with high neutron capture cross-sections [7,8]. In this context, boron stands out as an important material component in neutron shielding applications due to the high thermal neutron capture cross-section of the ^10^B isotope. In addition, the relatively low energy of the secondary radiation generated as a result of neutron–boron interactions is an important advantage that enhances the shielding performance of such materials [9,10,11].

Gypsum-based materials are one of the widely used binder systems in the construction sector and are especially preferred in interior applications due to advantages such as rapid setting, low density, good workability, smooth surface finish, and fire resistance. The main component of gypsum binder, calcium sulfate hemihydrate (CaSO_4_·½H_2_O), reacts with water and transforms back into the dihydrate form (CaSO_4_·2H_2_O), forming a solid and durable structure during this process. This hydration process directly determines the microstructure and mechanical properties of gypsum materials [12,13]. In addition, the low thermal conductivity of gypsum and its ability to absorb heat by releasing crystallization water during fire make it an important fire-resistant building material [14].

However, the mechanical strength and density of conventional gypsum materials may remain limited for advanced engineering applications such as radiation shielding. Therefore, in recent years, studies aimed at improving the physical, mechanical, and functional properties of gypsum matrices by adding different mineral additives have increased. Mineral additives play a decisive role in both strength and functional performance by altering the pore structure, crystal morphology, and density of the gypsum matrix [15,16,17].

In contrast to cementitious systems, gypsum-based matrices exhibit lower density and a distinct crystallization mechanism, which may lead to different radiation interaction behavior. Although numerous studies have investigated boron-containing additives in cement-based composites, studies specifically focusing on gypsum systems remain relatively limited, particularly in terms of integrated evaluation of radiation-shielding, mechanical, and thermal properties [15,16,17,18]. In this context, the novelty of the present study lies in the use of colemanite as a functional additive in gypsum-based composites and in the comprehensive evaluation of radiation-shielding, mechanical, thermal, and microstructural properties within a unified framework, which has been scarcely addressed in the existing literature.

In this context, boron-containing minerals attract attention, particularly due to the advantages they provide against neutron and gamma radiation. The ^10^B isotope of boron has a high thermal neutron capture cross-section and is effectively used in neutron shielding applications [10,19,20]. Among boron minerals, colemanite (Ca_2_B_6_O_11_·5H_2_O) stands out as an additive material due to its high boron content, natural availability, and compatibility with binder systems such as cement and gypsum [21,22,23]. In addition, the crystallization water and boron oxide components in the structure of colemanite provide properties that can contribute to both neutron moderation and gamma-ray attenuation [19,21,23].

The incorporation of colemanite into the gypsum matrix not only improves radiation-shielding performance but also leads to significant changes in the microstructure. This additive can affect the growth mechanism of gypsum crystals, promoting the formation of a denser and more compact structure, and accordingly, improvements in properties such as mechanical strength and density can be observed. However, depending on the additive ratio, negative effects such as increased porosity or reduction in the continuity of the binder phase may also occur. Therefore, determining the optimum additive ratio is of critical importance in terms of both mechanical and radiation-shielding performance.

As a result, colemanite-added gypsum composites are considered among next-generation building materials that have the potential to provide improved radiation-shielding properties while maintaining advantages such as lightness, workability, and economical production. Investigating the microstructural, mechanical, and radiation interaction properties of such composites together is of great importance for understanding the multifunctional performance of the material.

In this context, the main hypothesis of this study is that the incorporation of thermally treated colemanite enhances radiation attenuation performance through increased effective atomic number and boron content, while an optimum additive ratio exists due to the competing effects of microstructural densification and particle agglomeration.

This study aims to experimentally investigate the gamma and neutron radiation absorption capacity of colemanite-added gypsum-based composites. In this context, the linear attenuation coefficients of the prepared samples were determined, and the obtained results were evaluated in detail. In addition, the effects of colemanite addition on the radiation-shielding performance of the material were analyzed, and inferences were made to determine the optimum additive ratios.

It is aimed that the study will contribute to the development of safe, environmentally friendly, and economical building materials against radiation and help to eliminate the gap in the literature.

## 2. Materials and Methods

### 2.1. Materials

#### Materials Used in the Experiments

Colemanite (Ca_2_B_6_O_11_·5H_2_O), a boron-rich mineral, was used as an additive to evaluate its influence on gamma and neutron attenuation properties. Commercial construction gypsum supplied by ABS Alçı ve Blok Sanayi AŞ (Aşkale, Erzurum, Türkiye) was used as the matrix material.

The colemanite was obtained from the Bigadiç deposit (Balıkesir, Türkiye) and subjected to pre-treatment processes including crushing, drying, and sieving to achieve micron-scale particle size.

Prior to incorporation into the gypsum matrix, the colemanite was thermally treated in a muffle furnace at 650 °C for dehydration and impurity removal. The selected temperature is within the 600–700 °C range reported in the literature for effective removal of chemically bound water, resulting in a more stable and boron-enriched phase. This treatment is expected to enhance the radiation interaction efficiency and improve compatibility with the gypsum matrix [24,25].

### 2.2. Methods

#### 2.2.1. Production of Composite Material

The production of the composites examined in this study was carried out in the laboratories of the Faculty of Engineering at Erzurum Technical University. In this study, five types of materials were produced, including a standard gypsum sample and colemanite-modified composite materials.

First, a pure gypsum sample was prepared as a reference. Then, colemanite was added separately to each gypsum mixture at ratios of 1%, 2.5%, 5%, and 10% by weight. The water-to-binder ratio was fixed at 0.55 [26]. The temperature of the mixture was monitored using a digital thermometer, and the mixing water temperature was maintained within the range of 20–25 °C.

The prepared mixtures were cast into cube molds with internal dimensions of 5 cm × 5 cm × 5 cm. The samples were kept in the molds for 24 h and then demolded. During the curing process, the samples were stored in a constant temperature environment (24 °C), and ambient temperature and relative humidity were regularly monitored using a digital thermometer.

#### 2.2.2. Heat Treatment

Colemanite was subjected to a dehydration process in a muffle furnace at 650 °C for 6 h. After the heat treatment, the weight differences in the colemanite samples were measured, and the removal ratio of chemically bound water was calculated. According to the calculations, approximately 98% of the water content in the colemanite ore was removed [24].

According to the chemical analysis results, colemanite contains approximately 35.4% H_2_O [27,28]. After heat treatment, the water content was reduced to approximately 1%, and the material was used in the experiments in this form.

#### 2.2.3. X- and γ-Ray Attenuation Measurements

**Figure 1:**
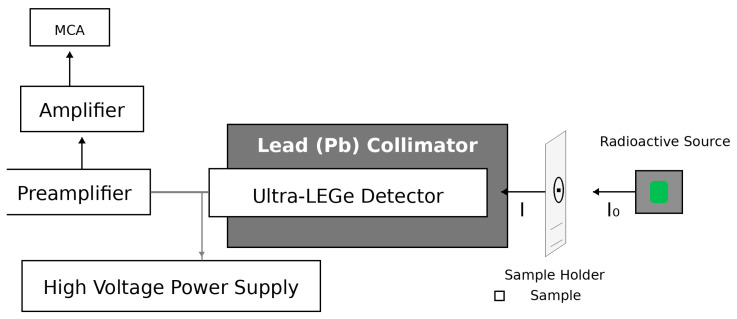
Experimental setup for gamma-ray attenuation. The green marker indicates the radioactive source position; colors are used only to distinguish setup components.

In this study, experimental measurements were conducted to determine the attenuation properties of the produced composite samples against X-rays and gamma rays. The measurements were carried out at the High Energy Spectroscopy Laboratory of the Department of Atomic and Molecular Physics, Faculty of Science, Atatürk University. The experimental setup used in the measurements is schematically shown in Figure 1. The setup consists of an HPGe detector with a 30 mm^2^ Ge crystal that is 5 mm thick. The detector has a 25-micron-thick Be window and a resolution of ~140 eV and 550 eV at 5.9 keV and 122 keV, respectively. The detector preamplifier was connected to a Tennelec 244 amplifier and an Ortec 926 analog-to-digital converter, and spectra were recorded on a PC using MAESTRO-32 MCA Emulation Software (version 6.08, ORTEC/AMETEK, Oak Ridge, TN, USA).

A variable energy VEX system and a Ba-133 radioisotope were used as radiation sources.

The initial intensity of a photon beam (*I*_0_) decreases exponentially as it passes through an absorbing material of thickness (*t*). This attenuation is expressed by the linear attenuation coefficient (LAC) defined within the framework of the Lambert–Beer law [5,29]. The linear attenuation coefficient was calculated using the following equation:

(1) $$\mu =-\frac{1}{t}ln\left(\frac{I}{I_{0}}\right)\;\;\;\;\;({cm}^{-1})$$

where (*I*) represents the intensity of the photon beam after passing through the absorber and (*I*_0_) represents the initial intensity before the material.

In addition, the ratio of the linear attenuation coefficient (*μ*) to the density (*ρ*) of the sample, defined as the mass attenuation coefficient (MAC), is a parameter independent of the physical state of the material and is widely used in radiation-shielding studies [29]. In this study, the mass attenuation coefficients were calculated using the following equation, considering the density values measured under the experimental geometry shown in Figure 1:

(2) $$\left(\frac{\mu }{\rho }\right)=-\frac{1}{\rho t}ln(\frac{I}{I_{0}})$$

where *ρt* is referred to as the mass thickness, and the unit of the mass attenuation coefficient is cm^2^/g [3,30].

#### 2.2.4. Neutron Absorption Measurements

The neutron measurements in this study were carried out in the Prof. Dr. Gökhan Budak Neutron Measurement Laboratory of the Department of Physics at Atatürk University.

Neutron absorption experiments were conducted using a ^241^Am–Be neutron source. Neutron detection was performed using a ^10^B-enriched BF_3_ gas-filled proportional counter (NP-100B, Canberra Industries, Meriden, CT, USA), coupled with an ADM-606M transportable ratemeter/data acquisition unit (Canberra Industries, Meriden, CT, USA).

**Figure 2:**
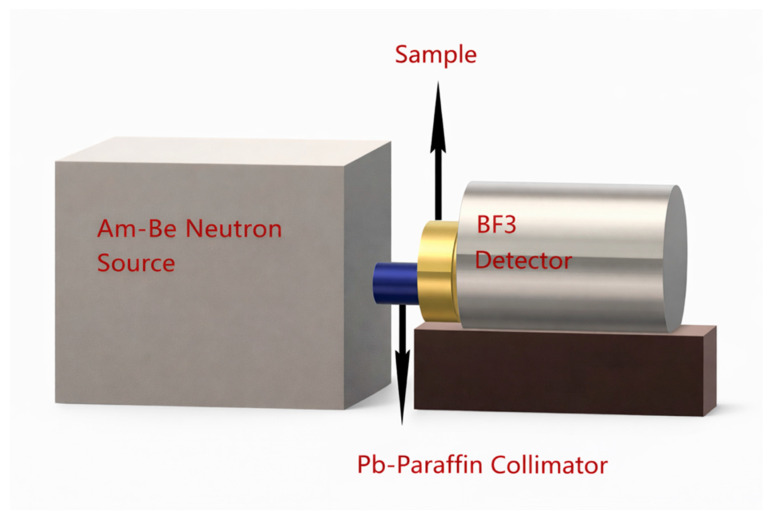
Experimental setup for neutron attenuation experiments.

The neutron attenuation behavior was evaluated using an exponential attenuation model analogous to the Lambert–Beer law:

N = N_0_ e^(−Σt)^ (3)

where N_0_ represents the initial neutron intensity, N is the transmitted neutron intensity after passing through the shielding material, Σ denotes the macroscopic cross-section (cm^−1^), which accounts for the probability of neutron scattering and absorption within the material, and t is the sample thickness [1,5,8]. It should be noted that, due to the polychromatic nature of the ^241^Am–Be neutron source, the macroscopic cross-section (Σ) used in this study represents an effective, spectrum-averaged removal cross-section, rather than a strictly monoenergetic interaction parameter. Therefore, the applied model provides a comparative and phenomenological description of neutron attenuation rather than an exact energy-dependent representation. The experimental setup used for neutron attenuation measurements is shown in Figure 2.

#### 2.2.5. Compressive Strength Test

The compressive strength tests of the produced composites were carried out in the Civil Engineering Laboratory of Erzurum Technical University.

**Figure 3:**
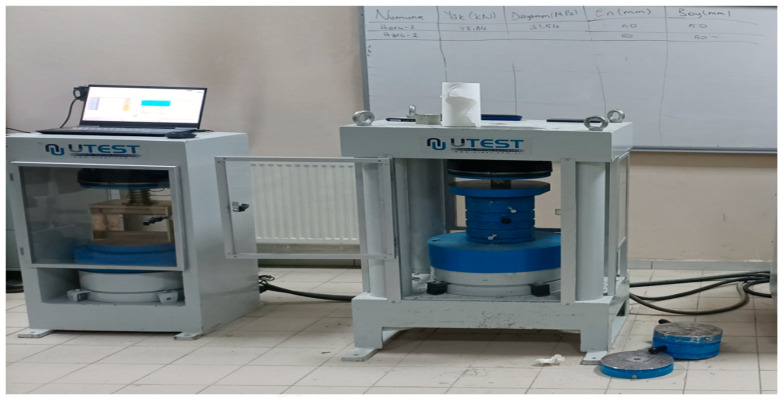
Compressive strength testing apparatus used for the gypsum-based composite specimens.

The experimental studies were conducted in accordance with the TS EN 12390-3 (2019) standard [31]. The samples were subjected to a constant loading rate until the maximum load capacity was reached. The test specimen was placed between the loading plates of the testing machine, ensuring that it was positioned perpendicular to the casting direction. The maximum load value was recorded, and the compressive strength was calculated using the following equation:

σ = P/A(4)

where σ represents compressive strength, P is the maximum load at failure, and A is the cross-sectional area subjected to loading [18,32,33]. The compressive strength testing apparatus is shown in Figure 3.

#### 2.2.6. Scanning Electron Microscopy (SEM)

SEM/EDX analyses were performed at the High Technology Application and Research Center (YUTAM) of Erzurum Technical University using a Quanta 250 FEG scanning electron microscope (FEI Company, Hillsboro, OR, USA).

#### 2.2.7. FTIR Measurement Procedure

FTIR analyses were carried out at the High Technology Application and Research Center (YUTAM) of Erzurum Technical University, using a Shimadzu IRTracer-100 spectrometer (Shimadzu Corporation, Kyoto, Japan).

#### 2.2.8. Thermal Conductivity Measurements

Thermal conductivity measurements were carried out in the Heat Transfer Laboratory of the Department of Mechanical Engineering, Faculty of Engineering and Architecture at Erzurum Technical University. Measurements were taken using a Linseis THB-100 Transient Hot Bridge thermal conductivity analyzer (Linseis GmbH, Selb, Germany).

The operating principle of the device is based on the determination of temperature distribution within the material under steady-state heat flux boundary conditions. For each sample, measurements were performed under controlled conditions, and the thermal conductivity values were calculated based on the recorded temperature gradients.

The measurement range of the apparatus is suitable for typical construction materials exhibiting moderate thermal conductivity. In order to ensure steady-state conditions, a stabilization period of approximately 20–30 min was allowed prior to data acquisition. For each specimen, three independent measurements were conducted to enhance the reliability of the results, and the reported values correspond to the arithmetic mean of these measurements.

Taking into account the sensitivity, calibration characteristics, and repeatability of the device, the overall experimental uncertainty of the thermal conductivity measurements was estimated to be within the range of ±3–5%.

## 3. Results

Gypsum and Gypsum composites containing dehydrated colemanite at proportions of 1%, 2.5%, 5%, and 10% were investigated. The X-ray and gamma-ray attenuation values of these samples are presented below.

### 3.1. X- and Gamma (γ)-Ray Attenuation Experiments of Colemanite-Added Gypsum-Based Composites

**Figure 4:**
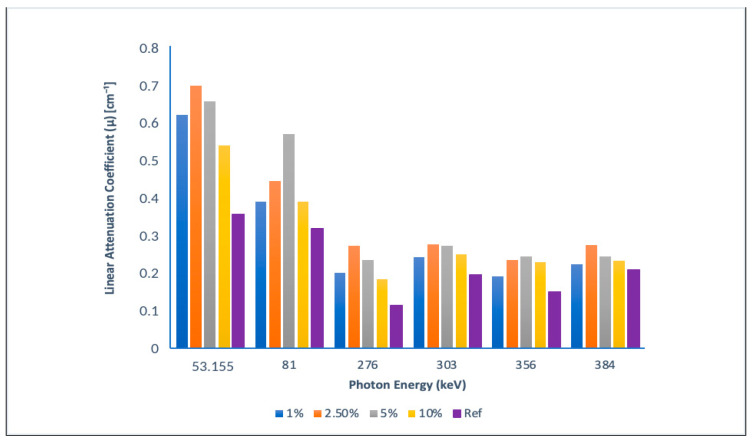
Linear attenuation coefficient (LAC) values of colemanite-added gypsum-based composites.

**Figure 5:**
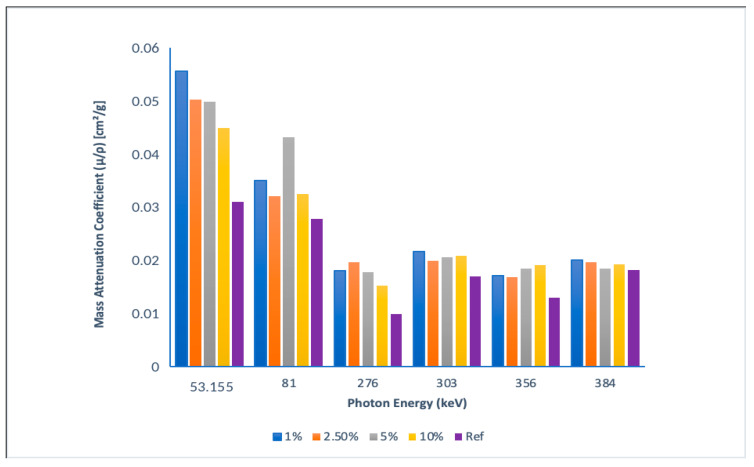
Mass attenuation coefficient (MAC) values of colemanite-added gypsum-based composites.

The X- and γ-ray attenuation results of the samples belonging to the prepared mixtures are listed in Table 1 and presented graphically in Figure 4 and Figure 5.

In this study, the attenuation behavior of colemanite-added gypsum-based samples at different photon energies was investigated, and the effect of the additive ratio on radiation-shielding performance was evaluated. The obtained findings indicate that the samples containing colemanite exhibit higher values in terms of both the linear attenuation coefficient (μ) and the mass attenuation coefficient (μ/ρ) compared to the pure gypsum sample.

This result can be explained by the change in the chemical composition of gypsum with the addition of colemanite. Compared to pure gypsum, colemanite-added gypsum forms a composite structure consisting of a combination of different elements. As a consequence of this modification, the effective atomic number and electron density of the material increase relative to pure gypsum, thereby enhancing the probability of photon interaction with the material.

In the low-energy region (53–81 keV), a significant increase in attenuation coefficients was observed. In this energy range, the dominant interaction mechanism is the photoelectric effect, which strongly depends on the effective atomic number of the material. Therefore, colemanite-added gypsum exhibits higher attenuation performance compared to pure gypsum in this energy range.

At intermediate energy levels (≈276 keV), although a general decreasing trend in attenuation coefficients is observed, it was determined that colemanite-added samples still maintain higher values compared to pure gypsum. This indicates that the addition of colemanite continues to enhance the interaction probability within this energy range.

In the high-energy region (303–384 keV), the differences between the samples were observed to diminish. This behavior can be attributed to the dominance of Compton scattering as the primary interaction mechanism. In this energy range, attenuation depends more on the electron density than the chemical composition of the material; therefore, the improvement provided by colemanite addition remains relatively limited.

Based on the evaluation of different additive ratios, it was determined that the gypsum sample containing 5% colemanite generally provides the highest attenuation performance, while the 2.5% additive ratio also offers a similarly high and balanced performance. The relative decrease in performance observed at the 10% additive ratio is thought to be associated with changes in the microstructural properties of the material.

In conclusion, the findings obtained in this study are consistent with the literature, indicating that the addition of colemanite improves the radiation-shielding properties of gypsum. This improvement becomes more pronounced for low- and medium-energy photons compared to pure gypsum. The optimum additive ratio is considered to lie within the range of 2.5–5%. However, it is also understood that the additive ratio must be optimized, as microstructural drawbacks at higher concentrations may limit the overall performance [21,22,34,35].

### 3.2. Neutron Attenuation Experiments of Colemanite-Added Gypsum-Based Composites

**Figure 6:**
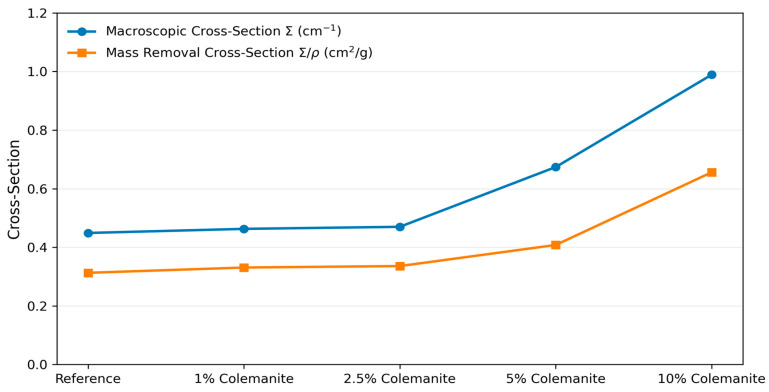
Neutron absorption of colemanite-added gypsum-based composites.

The neutron attenuation performance of gypsum-based composites incorporating different proportions of colemanite is presented in Table 2 and Figure 6. The experimental results reveal a clear and systematic enhancement in neutron shielding capability with increasing colemanite content.

A gradual decrease in the transmitted neutron intensity ratio (I/I_0_) is observed, decreasing from 0.956 for the reference sample to 0.906 for the specimen containing 10 wt.% colemanite. This reduction indicates a progressive attenuation of neutron flux through the composite structure as the additive ratio increases. Correspondingly, the macroscopic cross-section (Σ) values exhibit a consistent upward trend, increasing from 0.449 cm^−1^ in the reference sample to 0.989 cm^−1^ at 10 wt.% colemanite, representing more than a twofold enhancement. A similar trend is evident for the mass removal cross-section (Σ/ρ), which increases from 0.313 cm^2^/g to 0.656 cm^2^/g, further confirming the improvement in neutron attenuation efficiency.

The observed enhancement in shielding performance can be primarily attributed to the high boron content of colemanite, particularly the presence of the ^10^B isotope, which possesses a significantly high neutron capture cross-section. As the colemanite content increases, the probability of neutron interaction within the material correspondingly rises, leading to more effective absorption. In addition, the gypsum matrix, containing hydrogen-rich phases, contributes to the moderation of fast neutrons via elastic scattering, thereby increasing the likelihood of subsequent capture by boron atoms. This synergistic effect between neutron moderation and absorption plays a crucial role in the overall shielding behavior of the composites.

It is noteworthy that the increase in attenuation performance becomes more pronounced beyond 5 wt.% colemanite content, suggesting that a critical concentration of boron is required to significantly influence neutron interaction mechanisms. Unlike gamma radiation shielding, where an optimum additive ratio is often observed due to competing microstructural effects, the neutron attenuation behavior in this study demonstrates a monotonic improvement with increasing additive ratio, indicating that a higher colemanite content is advantageous for neutron-shielding applications.

In conclusion, the results clearly demonstrate that colemanite incorporation substantially enhances the neutron attenuation capacity of gypsum-based composites. These findings are consistent with the established understanding in the literature that boron-containing materials are highly effective for neutron shielding due to their combined moderation–absorption mechanism, thereby confirming the potential of colemanite as a functional additive in radiation protection materials.

### 3.3. Uniaxial Compressive Strength Test of Colemanite-Added Gypsum-Based Composites

**Figure 7:**
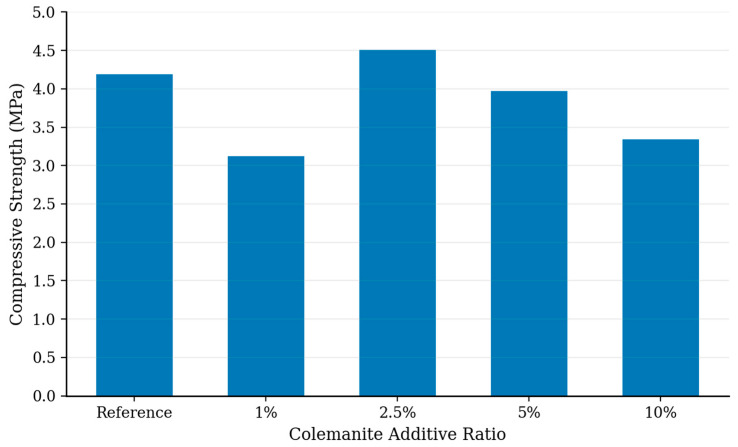
Compressive strength (MPa) according to mixture type.

After a curing period of 28 days under saturated water conditions, three specimens from each mixture were subjected to compressive strength testing, and the average values of these three specimens were calculated. The compressive strength values of the samples are listed in Table 3 and presented graphically in Figure 7.

According to the obtained results, it is clearly observed that the colemanite additive ratio has a non-linear effect on mechanical strength. While the compressive strength of the reference sample was determined as 4.19 MPa, it was found that this value varied depending on the additive ratio in the modified samples.

At a low additive ratio (1%), a significant decrease in strength (3.12 MPa) was observed. This behavior can be attributed to the insufficient homogeneous distribution of colemanite within the matrix or inadequate adhesion between the additive and the binding phase. Furthermore, the crystalline structure of colemanite may have led to the formation of weak interfacial regions within the matrix, resulting in reduced mechanical strength.

In contrast, the sample containing 2.5% colemanite exhibited the highest compressive strength (4.51 MPa), indicating that the optimum additive ratio lies within this range. This improvement can be associated with the filler effect of colemanite, which enhances the pore structure and contributes to the formation of a denser microstructure. Additionally, the appropriate additive ratio may improve matrix–phase interaction, thereby positively influencing load transfer mechanisms.

However, when the additive ratio was increased to 5% and 10%, the compressive strength values showed a decreasing trend again (3.97 MPa and 3.34 MPa, respectively). This indicates that excessive colemanite content disrupts matrix continuity and reduces the effectiveness of the binding phase. High additive levels may lead to particle agglomeration and an increase in microcracks, which adversely affect mechanical performance.

The findings clearly reveal a strong and meaningful relationship between microstructural characteristics and mechanical performance. At low additive levels (1%), the insufficient interfacial adhesion between the matrix and the additive phase results in reduced strength. In contrast, at 2.5% colemanite content, a more homogeneous and compact microstructure is formed, improving load transfer mechanisms and leading to maximum strength values. However, increasing the additive ratio to 5% and 10% promotes agglomeration within the matrix and increases porosity, and these structural deteriorations result in a noticeable decrease in mechanical strength.

This study demonstrates that colemanite addition has a significant influence on the mechanical properties of gypsum-based composites, and determining the optimum additive ratio is critical for material performance. In particular, the 2.5% addition provides the most favorable mechanical properties, whereas higher ratios lead to strength losses due to deterioration of the matrix structure.

### 3.4. SEM Analysis and Microstructural Evaluation

Scanning Electron Microscopy (SEM) analyses enabled a comparative evaluation of the microstructural characteristics of the reference and colemanite-added samples. The obtained images reveal significant differences among the samples in terms of crystal morphology, phase distribution, and porosity.

In the SEM images of the reference sample, needle-like (acicular) crystals formed within the gypsum-based matrix exhibit an irregular and heterogeneous distribution. The presence of distinct voids and micro-pores between the crystals indicates that the structure possesses a low-density and weakly bonded microstructure. Furthermore, the limited contact between crystals reduces the efficiency of load transfer mechanisms, resulting in relatively low mechanical strength.

In contrast, a significant modification of the microstructure was observed in the colemanite-added samples. Particularly in the samples containing 2.5% colemanite, the crystals form a more regular, homogeneous, and densely packed structure. It is evident that the acicular crystals are more effectively interlocked and that the intercrystalline voids are reduced. This indicates the formation of a more compact and dense microstructure, which is consistent with the observed increase in mechanical strength.

High-magnification SEM images further reveal that colemanite particles are distributed within the matrix, creating a filler effect and contributing to the partial filling of pores. This enhances microstructural continuity and strengthens the interfacial bonding between crystals. As a result, load transfer mechanisms become more effective, leading to improved mechanical performance.

**Figure 8:**
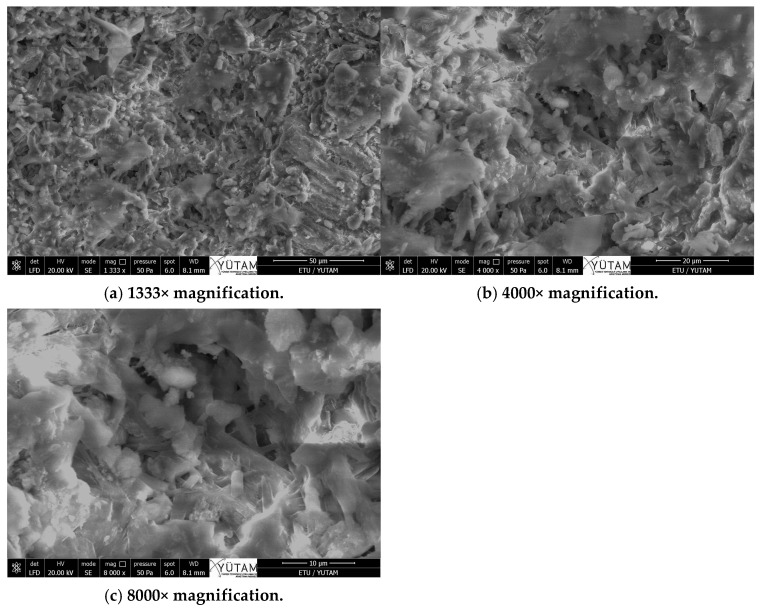
SEM images of the reference sample at different magnifications (1333×, 4000×, and 8000×). Needle-like crystal structures, irregular distribution, and a distinctly porous microstructure are observed.

**Figure 9:**
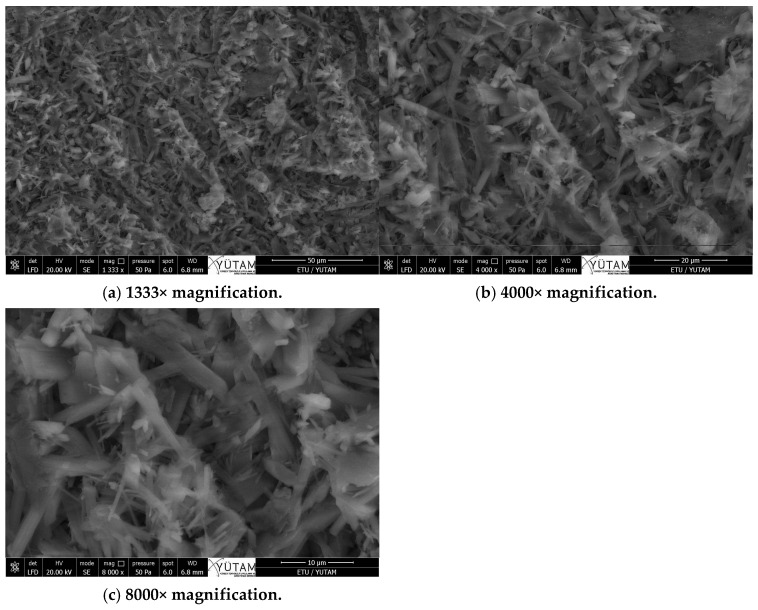
SEM images of the sample containing 2.5% colemanite at different magnifications (1333×, 4000×, and 8000×). A more compact microstructure, homogeneous crystal distribution, and reduced porosity are observed.

However, localized agglomeration regions were also observed in certain areas. It is considered that this phenomenon becomes more pronounced with increasing additive content and may lead to heterogeneity within the matrix structure. Agglomeration and the associated formation of voids can weaken the integrity of the microstructure, thereby causing a reduction in mechanical properties. Representative SEM images supporting these microstructural observations are shown in Figure 8 and Figure 9 for the reference and 2.5% colemanite-containing samples, respectively.

In conclusion, SEM analyses demonstrate that colemanite addition has a beneficial effect on the microstructure, leading to a denser, more homogeneous, and mechanically stronger structure, particularly at optimal additive ratios. These findings are consistent with the mechanical test results and clearly support the relationship between microstructure and mechanical properties.

### 3.5. FTIR Analysis and Spectral Evaluation

Fourier Transform Infrared Spectroscopy (FTIR) analyses were conducted to determine the chemical bonding structures and functional groups of the reference and colemanite-added samples. Examination of the obtained spectra reveals that the characteristic bands specific to the gypsum-based structure are preserved in both samples; however, partial changes in band intensities and positions were observed with the addition of colemanite.

The broad band observed in the range of approximately 3400–3600 cm^−1^ corresponds to O–H stretching vibrations associated with the crystalline water present in the structure. Similarly, the bands identified in the range of 1600–1700 cm^−1^ are attributed to H–O–H bending vibrations of water molecules. The presence of these bands in both samples indicates that the hydrated nature of the gypsum structure is maintained.

The strong absorption bands observed in the range of approximately 1100–1150 cm^−1^ correspond to the S–O stretching vibrations of sulfate groups (SO_4_^2−^) in the gypsum structure. In addition, the bands detected in the range of 600–700 cm^−1^ are associated with the bending vibrations of these sulfate groups. These characteristic bands demonstrate that the fundamental chemical structure of the gypsum matrix is largely preserved after the addition of colemanite.

When the spectrum of the colemanite-added sample is examined, an increase in band intensity and slight shifts are observed, particularly in the range of 900–1200 cm^−1^. This behavior indicates the contribution of vibrations associated with boron–oxygen (B–O) bonds present in the structure of colemanite. Since boron-containing compounds typically exhibit characteristic vibrations in this region, the observed changes confirm the successful incorporation of colemanite into the matrix [36,37,38].

Furthermore, the increased prominence of certain bands in the colemanite-added sample suggests that the additive enhances interactions within the matrix and induces partial modifications in the chemical bonding environment. However, the absence of entirely new bands indicating the formation of a new phase suggests that colemanite is incorporated into the gypsum matrix mainly through physical and partially chemical interactions.

**Figure 10:**
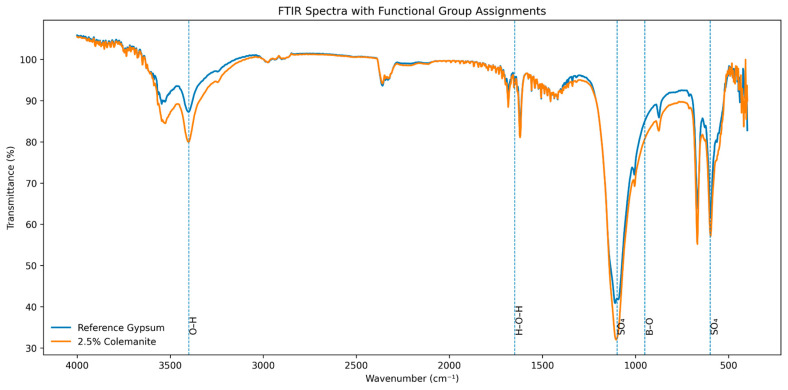
FTIR analysis results of gypsum and gypsum containing 2.5% colemanite.

The FTIR analysis results demonstrate that the addition of colemanite does not disrupt the fundamental chemical structure of gypsum-based composites but exerts a modifying effect on the existing bonding structure. In particular, the spectral contribution of boron-containing structures confirms the integration of colemanite into the matrix, while the preservation of characteristic bands associated with the hydrated structure and sulfate groups indicates that the structural integrity of the composite is maintained. These findings are consistent with the observed mechanical and microstructural improvements and support the conclusion that colemanite is an effective additive for enhancing material performance when used at appropriate ratios. The FTIR spectra of the reference gypsum and the 2.5% colemanite-containing gypsum sample are shown in Figure 10.

### 3.6. Thermal Conductivity Properties

**Figure 11:**
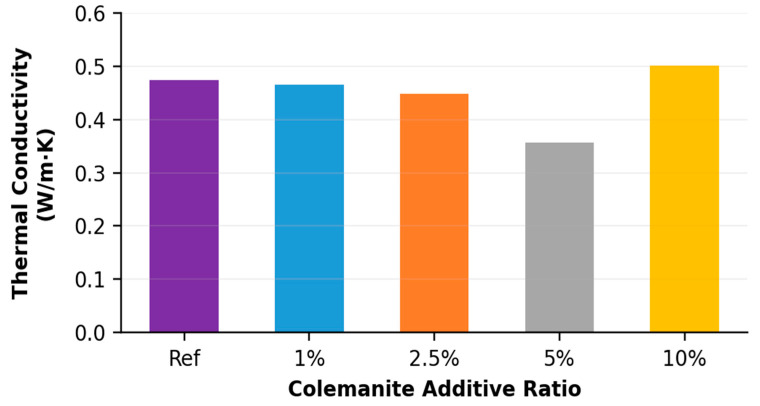
Thermal conductivity values (W/m·K) of gypsum-based composites containing different proportions of colemanite.

The thermal conductivity results of the colemanite-modified gypsum-based composites are presented in Figure 11. The findings reveal a non-linear dependence of thermal conductivity on colemanite content, indicating that the additive exerts a complex influence on heat transfer mechanisms within the composite structure. It should be noted that the non-linear and partially inconsistent trends observed between compressive strength and thermal conductivity at low colemanite contents (≤2.5 wt.%) arise from the different governing mechanisms of these properties. While thermal conductivity is mainly controlled by microstructural heterogeneity and phonon scattering, compressive strength depends on particle dispersion, interfacial bonding, and load transfer efficiency. Therefore, the lack of direct correlation at low additive levels is attributed to the dominance of different microstructural factors affecting each property.

The reference sample exhibits a thermal conductivity of 0.474 W/m·K, while a slight reduction is observed with the incorporation of 1 wt.% (0.465 W/m·K) and 2.5 wt.% (0.449 W/m·K) colemanite. This gradual decrease suggests that low levels of colemanite contribute to microstructural modification, likely introducing additional interfaces and heterogeneity that hinder phonon transport.

A pronounced decrease is observed at 5 wt.% colemanite, where the thermal conductivity reaches its minimum value of 0.357 W/m·K. This significant reduction can be attributed to the formation of a more porous and heterogeneous microstructure, which increases phonon scattering and disrupts continuous heat conduction pathways. The presence of microvoids and interfacial discontinuities at this composition likely plays a dominant role in suppressing thermal transport.

However, when the colemanite content is increased to 10 wt.%, the thermal conductivity rises sharply to 0.502 W/m·K, exceeding even the reference sample. This reversal in the trend indicates a transition in the governing heat transfer mechanism. At higher additive ratios, the increased solid fraction and potential particle agglomeration may lead to the formation of more continuous conductive pathways, thereby enhancing heat transfer. Additionally, the increased density and reduced effective porosity at this level may contribute to the observed increase in thermal conductivity.

Overall, these results demonstrate that the effect of colemanite addition on thermal conductivity is governed by the competition between porosity-induced thermal resistance and solid-phase conduction. While moderate additive levels (around 5 wt.%) optimize thermal insulation performance, higher concentrations promote structural densification and connectivity, leading to increased thermal conductivity.

From an application perspective, the 5 wt.% colemanite composition appears to be the most suitable formulation for thermal insulation, whereas higher additive ratios may be advantageous in applications where both radiation shielding and mechanical integrity are prioritized over thermal insulation.

## 4. Conclusions

In this study, the radiation-shielding, mechanical, microstructural, and thermal performance of gypsum-based composites modified with thermally treated colemanite were systematically and comprehensively evaluated. The results clearly demonstrate that the incorporation of colemanite induces a multifunctional enhancement in the composite system, significantly influencing its overall behavior across multiple performance domains.

The X-ray and gamma-ray attenuation results revealed that colemanite addition markedly improves shielding efficiency, particularly within the low- and medium-energy regions, owing to the increased effective atomic number and electron density associated with its boron-rich composition. At higher photon energies, the contribution of the additive becomes less dominant due to the prevalence of Compton scattering. Among the investigated compositions, the 2.5–5 wt.% colemanite range provides the most balanced and efficient gamma shielding performance, highlighting the existence of an optimal compositional window.

In contrast, neutron attenuation behavior exhibited a continuous and monotonic improvement with increasing colemanite content. The significant increase in macroscopic cross-section (Σ) and mass removal cross-section (Σ/ρ) values confirms the strong neutron absorption capability of the composites, primarily governed by the high capture cross-section of the ^10^B isotope. The synergistic interaction between neutron moderation within the gypsum matrix and subsequent absorption by boron phases constitutes an effective and integrated shielding mechanism.

Mechanical performance demonstrated a pronounced non-linear dependence on additive content. The maximum compressive strength was achieved at 2.5 wt.% colemanite, which can be attributed to enhanced particle–matrix interaction, improved packing density, and the formation of a more compact microstructure. However, higher additive ratios led to a deterioration in mechanical properties due to particle agglomeration, increased porosity, and disruption of matrix continuity. These observations were strongly supported by SEM analyses, which revealed a transition from a heterogeneous and porous structure to a dense and well-interlocked morphology at optimal compositions, followed by microstructural degradation at elevated additive levels.

FTIR analysis further confirmed that colemanite incorporation does not alter the fundamental chemical structure of the gypsum matrix but introduces boron-related bonding contributions, indicating successful integration of the additive at the molecular level.

The thermal conductivity results exhibited a complex and non-linear trend, governed by the competition between porosity-induced thermal resistance and solid-phase heat conduction. The lowest thermal conductivity was obtained at 5 wt.% colemanite, suggesting an optimal microstructural configuration for thermal insulation. At higher additive levels, the formation of more continuous conductive pathways led to a noticeable increase in thermal conductivity.

From a multifunctional perspective, the findings indicate that 2.5 wt.% colemanite provides the optimal balance between mechanical strength and radiation shielding, whereas 5 wt.% offers superior thermal insulation combined with high shielding efficiency. This highlights the critical importance of composition-driven optimization in the design of advanced composite materials.

From an architectural and building engineering standpoint, these composites offer a highly promising and versatile material solution for next-generation construction systems. Their combination of lightweight structure, enhanced radiation attenuation, and improved thermal insulation makes them particularly suitable for radiation-sensitive environments, such as medical facilities, nuclear infrastructure, laboratories, and specialized protective architectural spaces. Moreover, their compatibility with conventional gypsum-based construction techniques facilitates seamless integration into existing building practices.

In addition, the use of colemanite—a naturally abundant and environmentally benign boron mineral—provides a sustainable and cost-effective alternative to traditional high-density and toxic shielding materials, such as lead-based systems. The ability to tailor material properties through controlled additive ratios offers architects and engineers a flexible design strategy for multifunctional and performance-driven building materials.

Nevertheless, the observed mechanical and microstructural degradation at higher additive ratios underscores the necessity for careful optimization, particularly in structural and long-term applications. Future research should focus on durability performance, large-scale implementation, and the development of hybrid composite systems to further enhance the functional capabilities of these materials.

In conclusion, colemanite-modified gypsum composites can be considered as sustainable, multifunctional, and next-generation construction materials, capable of simultaneously meeting the demands of radiation protection, thermal insulation, and structural performance. These characteristics position them as a highly innovative and practical solution for advanced architectural and engineering applications.

## Figures and Tables

**Table 1:** Gamma absorption values of gypsum-based composites.

		Colemanite %1	Colemanite %2.5	Colemanite %5	Colemanite %10	Reference
E (keV)	I_0_	I	I	I	I	I
53.155	9548	7013	6746	6884	7297	7997
81	347,076	286,768	279,125	262,444	286,827	296,163
276	7019	6484	6257	6377	6539	6632
303	14,754	13,109	12,886	12,907	13,050	13,393
356	33,966	30,898	30,236	30,086	30,301	31,540
384	4135	3695	3605	3659	3680	3728

**Table 2:** Colemanite neutron absorption test values.

Sample	I	I/I_0_	t (cm)	M (g)	Rho (g/cm^3^)	Colemanite Additive Ratio (%)	Sigma (cm^−1^)	Sigma/Rho (cm^2^/g)
Reference	0.617272727	0.956	0.5	11.4153	1.4362	0	0.449	0.313
%1 Colemanite	0.616454545	0.955	0.5	11.107	1.3974	1	0.463	0.331
%2.5 Colemanite	0.616	0.954	0.5	11.1	1.3966	2.5	0.470	0.336
%5 Colemanite	0.60353	0.935	0.5	13.1368	1.6528	5	0.674	0.408
%10 Colemanite	0.58486	0.906	0.5	11.98	1.5073	10	0.989	0.656

**Table 3:** Average compressive strength values of the mixtures after 28 days of curing.

Sample Type	Average Load (kN)	Compressive Strength (MPa)
Reference	10.49	4.19
Colemanite 1%	7.80	3.12
Colemanite 2.5%	11.27	4.51
Colemanite 5%	9.92	3.97
Colemanite 10%	8.35	3.34

## Data Availability

The original contributions presented in this study are included in the article. Further inquiries can be directed to the corresponding author.
